# Functionality of Ingredients and Additives in Plant-Based Meat Analogues

**DOI:** 10.3390/foods10030600

**Published:** 2021-03-12

**Authors:** Konstantina Kyriakopoulou, Julia K. Keppler, Atze Jan van der Goot

**Affiliations:** Food Process Engineering, Wageningen University, P.O. Box 17, 6700 AA Wageningen, The Netherlands; julia.keppler@wur.nl (J.K.K.); atzejan.vandergoot@wur.nl (A.J.v.d.G.)

**Keywords:** plant protein, meat analogues, vegetarian sausage, vegetarian burger, vegetarian steak, binders, flavours, colourants

## Abstract

Meat analogue research and development focuses on the production of sustainable products that recreate conventional meat in its physical sensations (texture, appearance, taste, etc.) and nutritional aspects. Minced products, like burger patties and nuggets, muscle-type products, like chicken or steak-like cuts, and emulsion products, like Frankfurter and Mortadella type sausages, are the major categories of meat analogues. In this review, we discuss key ingredients for the production of these novel products, with special focus on protein sources, and underline the importance of ingredient functionality. Our observation is that structuring processes are optimized based on ingredients that were not originally designed for meat analogues applications. Therefore, mixing and blending different plant materials to obtain superior functionality is for now the common practice. We observed though that an alternative approach towards the use of ingredients such as flours, is gaining more interest. The emphasis, in this case, is on functionality towards use in meat analogues, rather than classical functionality such as purity and solubility. Another trend is the exploration of novel protein sources such as seaweed, algae and proteins produced via fermentation (cellular agriculture).

## 1. Introduction

The consumption of plant-based protein foods as a replacement for meat in western countries seems to encounter several barriers, despite the consumers’ awareness over environmental issues [1]. Among the barriers is the unwillingness of consumers to make this dietary change, due to the enjoyment of eating conventional meat, the nutritional and sensory appeal, as well as, the convenience that meat offers [2,3]. The development of protein-rich plant-based products with the potential to replace meat in the nutritional sense is already explored traditionally with the production of tofu, tempeh, seitan, etc. Recent meat analogue research and development focuses on the production of sustainable products that recreate conventional meat, not only nutritionally, but also in all of its physical sensations including texture, appearance, smell and taste [4]. Respective products that are available on the market are strips, chunks, patties and burgers, chicken-like blocks, ground beef-like products, nuggets, steaks, sausages, etc. 

Currently, technologies such as extrusion, shearing, spinning, and freeze alignment are employed or proposed for texturizing vegetable proteins from oilseeds, pulses and grains, forming a variety of structures, while fermentation has been used for many years now for the growth of mycoproteins (Quorn). Among these technologies, extrusion and mixing are mostly used in industry, with the rest being in a developing phase. Several recent reviews are summarizing the different structuring technologies, ranging from bottom-up-approaches such as preparation of individual fibres that are assembled to meat like structures (like in the case of mycoproteins), or top-down approaches, where the dough is formed into structured products (for example through extrusion) [5,6,7]. Less well summarized is the fact that the type of structure achieved is dependent on the functional properties of the ingredients used. It is therefore relevant to have a closer look at the ingredients used in currently available meat analogues. 

A typical plant-based meat analogue contains, apart from protein in textured and non-textured form, a significant amount of water, flavourings, oil or fat, binding agents and colouring agents. However, most of the ingredients used in those products are highly refined. That is the reason that meat analogues face more and more criticism for being artificial products [8]. The use of less refined ingredients is a development that gains traction in recent years [9,10,11]. To maintain new innovation in meat analogues, it is imperative to understand the role of each ingredient (refined or not) and their interplay to find alternatives that are better appreciated by consumers. 

In this overview, a first introduction is given to the different types of plant-based meat analogues produced through top-down technologies. This is followed by a discussion of the key ingredients of plant-based meat analogues. Special focus is put on the protein sources, and how origin and extraction methods affect their exerted functionality. We elaborate on the requirements for the development of different categories of meat analogues and we report cases and examples where specific ingredients could be used for the development of new meat analogues. 

## 2. Meat Analogue Products Formulation

Consumer preference studies in Western countries have shown that meat-eaters are more willing to switch to plant-based meat analogues when the products mimic meat in texture and sensorial properties and can be incorporated in a meal context that fits with the consumer’s expectations [12,13]. Focusing on this consumer segment more and more companies have launched plant-based products on the market resembling meat. The meat analogue product categories addressed in this study are minced products (burger patties and nuggets), muscle-type products (chicken or steak-like cuts) and emulsion-type products (such as sausages). The following sections summarize the characteristics of these products, as well as their requirements in terms of ingredients and additives. In Section 3, we provide information on how these requirements can be fulfilled by the currently available plant-based ingredients.

### 2.1. Emulsion-Type Products

Plant-based products such as sausages, frankfurters, bologna, mortadella, etc. are examples of emulsion-type products. Similarly to the animal-based formulation, plant-based products consist of a substantial amount of water, proteins, fats, carbohydrates (gums, fibres, starch, etc.), salt and spices. The inspiration for the formulation of many plant-based emulsion-type products comes from meat and meat extender applications, where high protein non-meat substances partially replace meat [5].

In animal-based emulsion-type products, finely chopped meat from different sources (pork, beef, mutton, etc.) is used to form a stable mixture that binds water and traps fat, giving the product its characteristic texture when cooked [6]. Depending on the types of meat used, a variety of products is produced ranging from high-quality all-meat sausages to economy-style sausages, where lower meat quality cuts are augmented with higher fat levels [7]. Although different parts of the animal are used in sausage production, lean meat is usually separated firstly and mixed with water and salt to maximize protein extraction. Afterwards, it is blended with the rest of the fat meat, spices and binders for the final formulation [14,15].

Lean meat contains soluble myofibrillar proteins with desired water-holding capacity and emulsifying properties [7]. Based on this, it can be expected that plant-based proteins with similar functionality as myofibrillar proteins in terms of solubility, water-holding and emulsification capacity can be a suitable replacement. Similar to their meat counterparts, plant-based emulsion products can comprise multiple emulsion systems (mostly W/O/W). However, next to good emulsification capacity, the plant-based ingredients used should present the ability to form coherent and strong gels. 

*Proteins*: Multiple plant proteins have the function of binding water, stabilise emulsions and gels, among which are soy protein, gluten, pea proteins, potato proteins (see Section 3.1). To achieve a coarser texture in emulsion-type formulations, there is also the possibility to add proteins in a texturized form in order [16]. Though, proteins are often combined with non-protein binders or fillers such as polysaccharides (e.g., fibres and starches (see Section 3.2)). The addition of those ingredients originates from the fact that it is known already that the presence of plant proteins often leads to a reduction in gel formation/elasticity in cooked emulsion products [17]. In recent product formulations such as “mimic-würstel” and “mimic-mortadella”, the use of less refined ingredients such as bean protein flour, chickpea flour and wheat flour as well as tofu has been suggested [18]. Amongst others due to the use of these ingredient combinations, the dry matter content of the products is higher than their meat counterparts. That has effects on juiciness. 

*Binders*: Several soluble binders such as soy protein isolate, methylcellulose, carrageenan and modified starches are used in emulsion-type meat analogues. Their role is to improve the textural properties of the products, providing the desirable gelling and thickening (see Section 3.2). Additionally, they can contribute to emulsion stability, reducing oil leakage and purge loss. 

*Fats*: Fat is an essential component as it improves juiciness, tenderness, and overall palatability of the emulsion-type products. The stability of both moisture and fat binding in the highly hydrated gel protein matrix is important. For meat products, rind emulsions or fat pre-emulsions are used, aiming at fat stabilization, preventing any fat separation during cooking and coalescence of the fat on the surface of the product [14]. Similar requirements are also expected for the plant-based counterparts where plant oils and fats are used (see Section 3.3). The type of fat (fat with a high or low melting point) seems to be less important for finely ground sausages applications like frankfurters, whereas fats with higher melting points are used when manufacturing cooked coarse cutting sausages or emulsion-type products with fat inclusions like mortadella [15]. Stabilisation of the fat in such plant-based foods can be achieved with the selection of the plant proteins with good emulsification capacities or even the use of native oleosomes [19] (see Section 3.1). Next to that, the use of protein-rich ingredient where oil is still in its native oleosome structure can also form fat containing gels when heated, which can be suitable for this type of products [20].

*Others*: Colourants and spices are also added to the product to resemble meat emulsion-type products. Heat stable colourants colours are mainly used, but those can be of natural origin (see Section 3.4.1). For example, fermented rice flour and paprika oleoresins were used in plant-based bologna formulations (Smart Deli^®^ Bologna by Lightlife) to a typical pink colour. A variety of natural savoury spices and meaty aromas are available [21] and are selected according to the type of product the meat analogue mimics. Salt remains an important taste enhancer. However, when in contact with proteins, it affects their functionalities [22]. Current nutritional trends aim at reducing salt content and especially sodium, leading to some product formulation challenges, not only for sausage-types of products but also for comminuted products and whole-cuts.

### 2.2. Burgers, Patties and Nuggets

Plant-based products resembling ground and bound animal-based meat products, aim at recreating their distinct bite, chewiness, succulence and firmness. Animal-based burgers, patties and nuggets, consist mainly of proteins and fats and to a lesser extent of seasoning, salt and binders (such as wheat crumb, starches and fibres). Although in smaller quantities, salt changes the structure of proteins and toughens the products [23], while binders provide water and fat retention, and improve the texture and appearance of the product [24]. Plant-based comminuted products follow closely the recipes of the corresponding animal products. The majority of the protein components is first transformed into a meat-like fibre structure that resembles ground meat, known as textured vegetable protein (TVP), and then mixed with the rest of the ingredients for the final formulation. 

**Figure 1:**
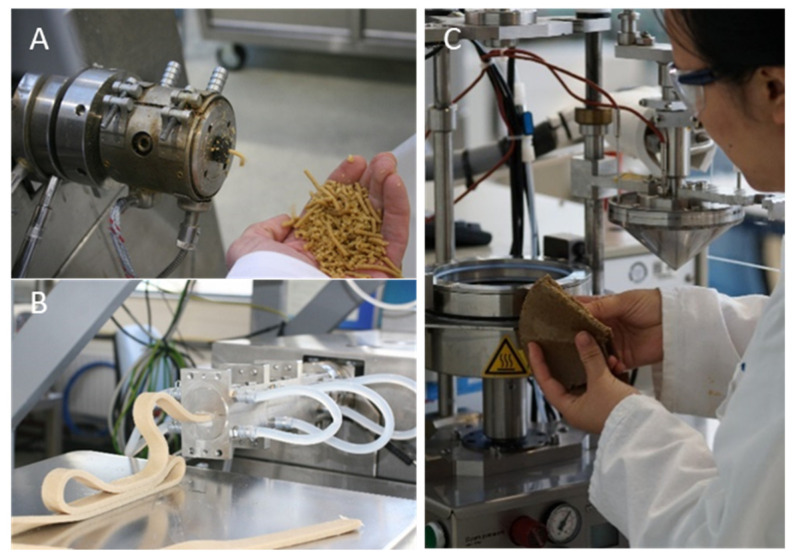
Technologies for plant protein texturization: (**A**) low moisture extrusion, (**B**) high moisture extrusion and (**C**) shear cell technology. The person in figure has given the consent to use this photo.

*Proteins*: The protein is often texturized using low moisture extrusion cooking (Figure 1A). The most prevalent TVPs used in meat analogues are those based on soy, wheat or pea protein and mixtures thereof. Nevertheless, there is an increasing number of protein sources that can be texturized and have the potential to be used for the development of new plant-based burger-type products (see Section 3.1). Hydrated TVP as an ingredient gives a meaty and chewy texture to the product and provides desirable juiciness in the final product formulation. Research is focused on the structuring potential of new protein sources and their ability to retain water during storage and to release it upon heating and deformation. However, TVP alone, similarly to ground meat, cannot form a coherent product, which makes the use of binders unavoidable.

*Binders*: Egg protein and methylcellulose are the main candidates in commercial products, while wheat gluten can also play this role as it creates a network when hydrated and helps bind the TVP and other ingredients together. The texture and mouthfeel of the products are further improved, with the use of texturizers that present high water holding moisture and can make the burger softer and juicier. For the latter ingredient requirements, protein isolates, protein concentrates and polysaccharides (see Section 3.2) can be used. 

*Fats*: The mouthfeel of juiciness is also affected by the fat in the product, which can be liquid or solid plant-based fat, emulsified or free (see Section 3.4). In many cases, a combination of liquid (such as sunflower and canola oil) and solid fats (like coconut or palm oils) is used to achieve the right balance (see for example the Beyond Burger^®^ and the Impossible™ Burger). Preferably the fats in the burger are solid at room temperature and turn liquid when the product is heated. This gives the product a pleasant mouthfeel, similar to corresponding meat products. 

*Others*: Moreover, “bleeding” vegetarian burgers attempt to create the feeling of juiciness by using beetroot juice and at the same time giving the product a characteristic meat colour (see Section 3.4). Research and development on plant-based burgers are furthermore focused on achieving even better juiciness and improving the appearance and taste of these products by developing new colour changing compounds, flavourings and aroma precursors.

### 2.3. Chicken-Like and Steak-Like Products

Another category of meat analogue products aims at mimicking whole-cut meats, like chicken meat, pork and beef steak, that are characterized by the presence of long fibres or layered structure. Plant-based products mimicking this fibrous or layered structure are produced via extrusion mainly. The products are processed further by freezing, curing, marinating and cooking to achieve the final structure, colour, tenderness, aroma and flavour change. So far, extrusion can be used to make small pieces of those products, while the shear cell technology (which is still in development) has the potential to make large pieces of fibrous plant-based products [25] (Figure 1B,C). The advantage of these products is that the desired structure of the final product is already there, so there is no need for reconstitution as it is required for the burger-type products. This can significantly reduce the ingredient list since binders and other texturizing agents can be omitted. However, this means that the structuring step is the key step in the formation of both a fibrous and a juicy product.

*Proteins*: Soy protein is the most commonly used ingredient. Not only isolated but especially less refined forms of soy protein such concentrates are used in extrusion applications [26,27]. With the use of an isolate, in several cases, additional components such as wheat gluten or carbohydrate fibres, are used for the formation of a multi-phase blend [28,29]. The mechanism of fibre formation is then based on the alignment of those phases [25]. Finally, solidification of the structure is achieved usually by cooling. The products take their shape based on the equipment used.

*Binders*: On the contrary to the previous meat analogues categories, binders are not necessary for whole-cut-type meat analogues due to the structuring technology used.

*Fats*: Fat is added to a limited extend during the structuring step, while the texturized products can be enriched with fat in a later stage (through marination). Liquid oils seem to be the preference of the industry for these types of products (see for example the vegan NoChicken chunks by The Vegetarian Butcher). However, there is still room for improvement, especially when the products are mimicking raw steak-like pieces where marbling effects maybe be desirable (see Section 3.3). Technologies to texturize vegetable fat are currently explored for this purpose [30].

*Others*: Colouring agents and flavours (including salt) can be either added during the structuring procedure or applied in the form of a marinade afterwards (for more details see Section 3.4), depending on whether the product mimics raw or cooked meat. 

Regarding the flavour addition, most applications are based on marination afterwards, since the conditions used inside an extruder are detrimental for flavour compounds. In a patent by Ojah, it is reported that the marination process through infusion is more successful if the wet extrudate product is first frozen and then thawed prior to diffusion [31]. This means that cooling and freezing steps after the extrusion process can be beneficial. 

## 3. Plant-Based Ingredients for Meat Analogues

In the following sections, different ingredients are described that are used or suggested for the production of the aforementioned categories of meat analogue products. We look into the role of bulk ingredients, such as proteins, oils and fat, and ingredients used in smaller quantities, such as binding agents, flavouring and colourants, on the desired texture and appearance of meat analogues. The required functionalities of the different types of meat analogues dictate which ingredients can be successful for each specific product development. Ingredients can have multiple properties and functionalities. Within this review, we discuss their purpose and end with suggestions for a route to create more natural plant-based products. 

### 3.1. Plant Proteins

Although the selection of plant protein ingredients is the starting point for product development, the actual choice is often dictated by the protein availability, the yield of the crops and the protein extraction potential. A common characteristic observed among the most frequently used plant-based ingredients is that they are originally by-products of the food industry (primarily oil or starch production). For example, soy meals collected after oil extraction were formerly used as animal feed. However, the high protein content, balanced amino acid composition, wide availability, low price and the specific protein functionalities of soy (such as good gelling properties and water holding capacity) [4], made way for the production of protein-rich food ingredients that are used in meat analogue applications. The popularity of soy however decreased in recent times in western countries [32] because of criticism on crop production (i.e., deforestation of the rainforest) [33] and potential negative health effects associated with the presence of specific antinutritional factors [34]. Protein from wheat, which contains a high amount of gluten, is another frequently used ingredient. Gluten has unique film-forming properties that result in small fibres when applied in meat analogues. Besides, it is cost-effective because the starch present in wheat flour is used industrially as well. The main drawback is that part of the population is intolerant to gluten. 

Apart from soy protein and gluten, other protein-rich oilseeds and leftovers from oil production are also considered as an ingredient for meat analogues, for example, sunflower and rapeseed meals. In addition to that, more and more crops are explored for their protein content, for example, rice, other cereal and bean flours. Derivatives of these crops such as meals, concentrates and isolates are used in traditional and novel meat analogues [4,35]. However, despite the increased interest in alternative protein crop, just meeting the demand for nutritional and functional characteristics is not enough; the socio-economic viability is also important. Lupin is an example of such a crop. Despite the huge potential and market demand for lupin-based ingredients/products, lupin cultivation in Europe remains largely insufficient to guarantee a steady supply to the food industry [36]. In the following sections, we look into currently used and promising protein ingredients for the three meat analogue categories: emulsion-, burger- and muscle-type products.

#### 3.1.1. Soy protein

Soybeans contain a mixture of water-soluble and insoluble proteins, of which the whole aqueous extractable proteins can be separated into storage globulin and whey fractions by acidification to pH 4.5–4.8. The extractable globular proteins are classified into four protein categories 2S, 7S, 11S and 15S according to their sedimentation coefficients. The 7S (β-conglycinin) and 11S (glycinin) fractions represent more than 80% of the proteins [37]. The protein content (type and ratios between different proteins) [38,39] and the presence of additional compounds (such as carbohydrates) [40,41] determine the functional properties of soy ingredients. For example, Tarone et al. (2013) found that gels produced at pH 3 of soy protein fractions rich in 7S exhibited higher stress at rupture and higher water holding capacity than those of rich in 11S [38]. In another research, the hydrophobic interactions of the proteins and thus the foamability and surface elastic behaviour of the soy proteins were affected by the presence of soyasaponin [40]. Moreover, the addition of external carbohydrates, such as inulin, is found to proportionally improve tofu hardness and enhance the incorporation of protein into the gel matrix [41].

Soy protein isolates and concentrates are the most commonly used ingredients for sausage-, burger- and meat muscle-like meat analogues. The production process of those ingredients determines the composition of the ingredients and their functionality. Both soy milk and defatted soy flour are used for making protein-rich ingredients. In the case of soymilk, produced by aqueous extraction of whole soybeans, concentration or spray drying is used to yield a powder of 45–50% protein content and ~30% fat [42,43]. Defatted soy flour with a protein content of about 50% [44], is used to make concentrates and isolate through a process known as fractionation. The concentrates specifically are extracted with aqueous alcohol or acid solvent resulting in final protein content of 70%. The isolates, which have a protein content of 90%, are produced using alkaline extraction followed by a precipitation step in acidic pH and neutralisation [45].

Regarding the protein functionality requirements for the different meat analogues (see Section 2), these can be achieved both by using highly refined and only enriched soy protein ingredients. Table 1 provides a summary of the functional quality as well as the application potential of different soy ingredient, as well as other protein-rich ingredients. Post-treatment processing, such as toasting or moisture heating [46,47], or even mixing with other protein or polysaccharides [48] is suggested for tuning the protein properties. For meat analogue applications, protein purity does not have to be so high. Stronger mixtures of soy protein isolate (SPI) and gluten or soy protein concentrates (SPC) have been used for the production of TVP based patties [4], while less refined ingredients have also been used for soy emulsions and gels for sausages [49,50], or for structuring muscle-like products [51,52,53,54].

#### 3.1.2. Wheat gluten

Wheat gluten is a key ingredient for many analogues. It has an attractive price for the industry as it is a by-product of bulky wheat starch production. In contrast to soy, extraction of gluten from wheat is done by washing out the soluble and dispersible components with water only, leaving behind the insoluble protein. Apart from binding and dough forming capacity, wheat gluten has additional desired functionality (including viscosity, swelling, nutritional quality) [55,56]. Its characteristic functionality allows it to be used both as a binder and as a structuring agent. The use of gluten in extrusion or shear cell can transform raw materials into fibrous structures, providing a base for both whole-cut and minced meat-types of analogues [57,58]. Gluten forms thin protein films upon simple deformation and elongation, transforming the meat analogue dough into a fibrous material [53]. This three-dimensional network is a result of disulphide protein linking [59], which causes also the formation of fibrous structures during high-moisture extrusion [26,60,61]. Disulphide bonds can be intramolecular or intermolecular, depending on the protein class. For gliadins, the low/medium molecular weight monomeric proteins, mostly intramolecular disulphide bonds will be formed, while for glutenins intermolecular disulphide bonds are more likely [62]. This means that gluten functionality is determined by the ratio of glutenins:gliadins. Isolation of specific protein subunits [63], modification of the protein during extraction (for example the use of non-reducing and reducing conditions [64] or hydrostatic pressure and temperature [65]), and interaction of gluten with other compounds such as polyphenols [66] and alkali salts [67] can lead to different degrees of cross-linking, which affect the eventual structure of the gluten in the meat analogues. Through hydrolysis other properties like solubility, foaming, and emulsifying qualities can be improved [68], broadening the application field of gluten. Current trends however are going towards replacing gluten due to its correlations with celiac diseases, although its unique properties are difficult to replace. Suggestions have been made to replace gluten with other types of proteins mimicking the properties of gluten after the addition of hydrocolloids, fermentation or hydrolysis [69,70]. The use of cross-linking enzymes such as transglutaminase or phenolic compounds and fibres with cross-linking potential is also an option for the modification of the alternative proteins [71,72,73].

#### 3.1.3. Legume proteins

Legume proteins from pea, lentil, lupine, chickpea, faba bean, mung bean and other types of beans have been examined on their functional properties, such as emulsification, foam stabilization and gel formation [74,75,76,77,78,79]. Among those plant proteins, pea protein has gained a lot of attention since it can be used in meat products and meat analogues in several forms based on final product formulation, the technology used, and any regulatory requirements [80]. The properties of the pea ingredients can be affected by the pea cultivar, the extraction process and the actual protein composition (legumin/vicilin ratio) [79]. The ability of pea ingredients to bind water and fat, and to generate a firm texture after thermal processing, allows them to act as binders, fillers, and functional improvers [80]. Structuring of pea protein by high moisture extrusion [81,82] and shearing [83] to produce meat analogue fibres has been successful. However, pea-based structures were found weaker than their soy-based counterparts [84,85]. This explains why often hydrocolloids are added in the case of pea protein-containing meat analogues.

Next to pea proteins, chickpea-, lentil-, faba bean-, mung bean- and lupine proteins also have good emulsion and foam stabilisation capacities [86,87,88,89,90,91], placing them as good alternatives to soy for sausage-type meat analogues. Unfortunately, proteins from lentil, lupine and faba bean present weaker gelling capacities than soy [84,92,93,94], limiting their application range. Mung bean and chickpea on the other hand present good gelling properties and are therefore more promising for meat analogues [75,86,87]. Recent research is focused on understanding and improving the functionality of ingredients (mostly isolates). For example, ultrasonication has been employed to improve gelling properties and thermal stability for legume proteins under optimized treatment conditions [95].

In case the ingredient after fractionation does not possess the desired functionality, it is possible to apply post-treatment steps to modify the protein composition and functionality. For example, pH shifting, the use of NaCl, calcium or high hydrostatic pressure during fractionation can change the protein structure, which in turn affects properties like gelation [93,96]. Thermal post-processing treatments of the ingredients, such as toasting, were also found a good way to modify legume ingredients, increasing their water holding capacity and decreasing their solubility [47,97]. An alternative is to use mixtures of ingredients to obtain the right functionality [98]. Mixing different legume proteins with polysaccharides or even using less-refined protein-rich ingredients can be a way to improve the functionality of legume proteins [99,100,101]. Vogelsang-O’Dwyer et al. (2020) showed that dry fractionated protein-rich faba bean flour exhibited superior functionality compared to isolate produced through acid extraction/isoelectric precipitation. The former showed higher protein solubility (~85%) at pH 7, increased foaming capacity and good gelling ability [102]. Therefore, many researchers look at the properties of mildly fractionated legumes, that are produced by milling and air classification [77,103,104,105,106,107].

#### 3.1.4. Rapeseed, sunflower and other seed proteins

In addition to legumes and soy, oilseeds such as rapeseed/canola, sunflower, and others have also gained interest. Although most seed isolates and concentrates are not commercially available yet, they seem to have promising functionality for replacing soy in plant-based food formulations. The fact that many of these proteins are by-products of the oil industry, makes their valorisation quite attractive economically. Limitations observed due to the presence of antinutritional factors or polyphenols that can react with the proteins and can hinder their application for human nutrition [108] are currently addressed by using extraction protocols [109,110], fermentation processes [111,112] or crop breeding [113]. Salgado et al. (2012) reported that sunflower protein concentrates have moderate water holding capacity values, similar to commercial soy protein isolates that are used as a thickening agent. Moreover, sunflower proteins are known to have good stability for emulsions and foams, comparable to commercial soybean protein isolates and bovine serum albumin [114]. Malik et al. (2017) report enhancement of the emulsification and the foaming capacity of sunflower protein isolates when the pH is kept near the isoelectric point (pH 4–5) while applying heat [115]. To act as an emulsifying agent, high protein purity was not a prerequisite according to Karefyllakis et al. (2019). They showed that mildly fractionated ingredients from sunflower containing three main classes of macromolecules (proteins, polysaccharides and oil bodies) exhibited satisfactory emulsification performance [116]. Moreover, sunflower protein concentrates also showed a gelation capacity comparable with commercial proteins used as gelling agents in different applications [114]. Lastly, the treatment of sunflower isolates with ultrasonication lead to the formation of stronger gels when applying a temperature of 95 °C. The firmness was further increased as the temperature decreased to 25 °C [117]. The protein heating causes the unfolding of proteins and the exposure of its hydrophobic groups, which allows the formation of non-covalent interaction among the denatured protein molecules reinforcing the colloidal network upon cooling [117].

Similarly to sunflower proteins, rapeseed proteins, consisting mainly of cruciferin (11S globulin) and napin (1.7–2S albumin), can gel under high pressure or heat and thus might promote meat-like textures [118,119]. However, differences in the properties of the rapeseed protein subunits have been reported; cruciferin forms a strong heat-set gel under alkaline conditions, while napin in general forms a weaker gel [120]. Ainis et al. (2018) reported that rapeseed proteins as a whole formed solid gels only at pH 7, while at pH 5 and pH 3 they had viscous-like behaviour after heating [121]. Other researchers report that gelation of rapeseed and canola proteins typically involves the addition of fixatives (e.g., transglutaminase), the chemical modification of the proteins (e.g., succinylation and acetylation), and the use of polysaccharide mixtures [122,123]. Apart from gelation, rapeseed proteins exhibit good emulsification and foaming properties [124,125], which can promote their application in sausage-type meat analogues. These properties are further improved even at alkaline pH by the presence of polysaccharides such as gum Arabic [126]. 

Quinoa seeds, belonging to the category of pseudo-cereals, have also gained attention as a protein source. Less refined quinoa ingredients such as whole seeds and flours have been encountered in meat products as meat extenders in nuggets [127], gelling agents in mortadella [128], fat replacers in burgers [129], and as binders and means to reducing nitrate/nitrite additions in sausage-type products [130,131]. Research is focused on understanding how quinoa protein (rich in 11S globulin and 2S albumin) behaves. Quinoa isolate presents average solubility (~50%) at neutral pH, however, its water absorption, emulsifying and foaming properties are similar to that of soy protein [132]. Good gelation properties are also reported [133,134], together with fibre-like connections in the gel network at low pH and divalent salt addition heat gelation procedures [135]. So far, there is limited research on the use of quinoa in extrusion processes (apart from low moisture extruded snacks). This suggests that quinoa protein is more suitable as an ingredient for sausage-type products and as a binding/thickening agent for TVP based products. 

Research on other seed proteins, such as chia and pumpkin seed proteins, is still in a pioneering stage and thus focused on protein isolation (including alkaline treatment and dry fractionation) and the resulting functionality [136,137,138,139]. For chia protein-rich ingredients, emulsifying stability was the highest for less refined fractions, while the protein-enriched fractions through dry fractionation exhibited better protein solubility, water absorption, foaming and least gelling capacity [136]. These properties indicate their potential to be used as an ingredient for emulsion-type products like sausages. Similar to chia seed protein, the solubility of pumpkin seed proteins is also rather low (<20%) in the acidic pH region (pH < 5), but it drastically increases at pH above pH 6 [140]. Pumpkin seed protein with denaturation temperatures above 90 °C seems to be more resistant than other proteins [139,140], which however might be the reason that these proteins lack important functionalities thus further modification should be taken into consideration [141].

#### 3.1.5. Other plant proteins (Peanut, Potato, Zein, Hemp)

Protein-rich streams such as peanuts, hemp, potato proteins and corn zein fractions are also promising sources of protein in food applications. Already in earlier research peanut flour was texturized using single-screw extrusion and presented functionality comparable to that of textured soya flour [142]. As potential ingredients for meat analogues, peanut protein concentrates showed comparable oil binding and foaming capacity to soy protein isolates. Moreover, they presented higher viscosity and gel formation after heating [143]. Rehrah et al. (2009) formulated a defatted peanut flour meat analogue with a protein content of 55%. The physicochemical and sensory properties of the product were similar to soy-based TVP, and the researchers suggested that it can be used for the development of ground beef like substitutes [144]. Recently, high-moisture extrusion products with a fibrous structure were also reported [145,146].

Proteins isolated from hemp seed meal (a by-product of the edible hemp oil industry) were found to exert a range of functionalities, regarding solubility, emulsification, water and oil holding capacity. These functionalities were affected by the extraction and isolation process [147,148,149,150]. An interesting property of hemp ingredients for meat analogue application is the least gelling concentration, which ranges from 12% w/w for the hemp meal to 22% w/w for the isolate [150]. Next to that, evidence was reported on the potential use of hemp protein concentrate for the substitution of SPI in high moisture extrusion meat analogues [151].

Potato proteins and zein, either separate or as a blend, can be used as meat analogue ingredients. Potato protein has good emulsification, foaming and gelation properties [152,153,154], making it a good texturizer. It consists of the majority from patatin and has a low thermal denaturation temperature (55–75 °C) at which it forms a gel network [154]. Thermally formed gels from potato protein isolates were obtained at pH 3 and pH 7 with minimal gelation temperatures around 45–50 °C [155], which can be beneficial for applications where a low temperature is required. However, similar to the rest of the proteins, potato protein properties can change with the use of different protein isolation methods [156] or post-treatment modification using enzymes [157].

Zein has been successfully used in stabilizing oil-in-glycerol emulsion-gels fortified with antioxidants as a healthy substitute for margarine in cake preparation, as a fat analogue in mayonnaise formulation [158], stabilization of foam and emulsions [159], and in the production of gluten-free bread or dough [160,161]. Glusac et al. (2018) reported gel-like structure formation of zein-potato protein stabilized used in meat analogues [162]. Recent research on the formulation of zein fibres by Mattice and Marangoni (2020) showed that individual fibres of the smallest scale can be produced by electrospinning. In addition, a web-like network can be produced by antisolvent precipitation, while an oriented fibrous network could be achieved by mechanical elongation. These zein fibres when incorporated into model meat analogue soy protein isolate gels, could potentially create texture mimicking chicken meat [163].

#### 3.1.6. Outlook of Plant Protein Usage for Meat Analogues

Soy, pea and gluten are so far the main ingredients encountered in commercial meat analogues. Those ingredients have in common that they are widely available, and are by-products of already established food /ingredient production lines, which goes along with the low cost. Research so far aimed at determining the most relevant functional properties for meat analogue applications, among which gelling and emulsification are mostly investigated. Generally stated the functional properties of readily available ingredients are not optimised for meat analogue applications, since their fractionation processes were designed before this application became important. It is therefore interesting to investigate options to tune the properties of these ingredients towards meat analogue applications, by blending ingredients (for example proteins with polysaccharides) or by inducing modification through post-treatments (inducing covalent interactions during processing for example [164]).

From the overview above we also see that many protein sources are explored and a broad range of properties are reported. Several promising protein ingredients however are not commercially available. Probably, the economic feasibility is hampered by the complexity of the fractionation process and the lack of use of any by-products of those novel crops. However, new crops can be used to develop fractionation processes that are aimed at meat analogue applications, for which that inclusion of non-protein components can be accepted, leading to reduced by-products stream. This means that more options are available to improve both functionality and resource use (see also Section 4). In addition, more insights on how the ingredients behave can help to find the right directions to improve the overall ingredient production process. 

### 3.2. Binding and Texturizing Agents 

The formulations of currently available meat analogues include several ingredients from animal or plant origins, which act as stabilizers, gelling agents, thickeners, emulsifiers. These ingredients can bind water and/or fat and can provide adhesion for the TVP particles. For sausage type products, binders are used to improve the smoothness and consistency of the product, and to maintain a juicy product by retaining the desirable moisture and fat. For meat emulsion products, phosphates (E450) and NaCl improve the binding properties of myofibrillar proteins [165,166], however, they are not well perceived by consumers that demand more natural ingredients [167,168]. Recent research on meat products has shown the potential of using citrus fibres [169] or fibre-rich fractions of cereals as alternatives binders for bologna-type products [131]. For non-vegan meat analogues, egg white or albumen can provide the desired functionality (used for example in The vegetarian butcher’s Little willies). Alternatively, the use of methylcellulose, carrageenan, locust bean gum, calcium alginate and other ingredients are encountered (see for example the Knacki Vegetale by HERTA (vegetarian product) and the Garden Gourmet Sensational sausage braadworst by Nestle (vegan product)). Arora et al. (2017) showed that mushroom-based sausage analogues containing 5% saturated fat and produced with carrageenan and xanthan gum exhibited improved textural properties (purge loss and emulsion stability) compared to those produced with soy protein concentrate and casein [170]. This improvement can be attributed to the gelling and thickening abilities of polysaccharides [171] and explains their increasing use as meat analogue binders and extenders. Despite the commercial application of polysaccharides in vegan products, the fact that E numbers or a long ingredient list with scientific equivalent names have to be labelled on the package [for exampleE461 (methylcellulose), E407 (carrageenan), E410 (locust bean gum), E412 (guar gum) and E415 (xanthan gum)] does not appeal to consumers. This has to do with the perception of consumers regarding their naturalness [172,173] and their impacts on health and wellness [174].

Clearly, the use of egg is not an option for vegan burger products. In those products, the requirements for binders are slightly different, however. Next to water and fat binding capacity, the binder helps to improve the texture and appearance of the burger by gluing the minced particles together. The functionality of hydrocolloids such as methylcellulose, hydroxypropyl methylcellulose, long fibre cellulose, corn zein and alginates, to bind texturized vegetable proteins, to improve oil encapsulation and to reduce oil absorption, is well documented [175,176,177,178]. Among these ingredients, methylcellulose is the most commonly used ingredient and can be found in many signature products of meat analogue companies despite its E number. Its binding capacity in combination with properties such as the unique reversible thermal gelation, the abilities to control ice crystals formation and reduced cooking loss [179,180,181], have made it a key ingredient for both comminuted products and emulsion type products. As a replacement of methylcellulose other hydrocolloids or combinations thereof can be considered. Alginate solutions can provide adhesion for particular materials [6] as in the presence of divalent cations they form a cold-set gel [182]. Additionally, enzymes like transglutaminase that induce crosslinks between the protein molecules can be used, improving the binding properties and the sliceability of finely-textured plant-protein products [6]. However, the use of enzymes is not perceived well from the consumers’ point of view due to its correlation with celiac disease [183]. Although enzymes, used as processing aids, may not have to be labelled when they are inactivated by cooking before final packaging [184], their usage does add to the costs of the ingredients. Oil can also play a binding role, especially at increased concentrations. Oil interacts with the proteins and promotes hydrophobic interactions and aggregation resulting in a uniform gel network [185]. However, despite the possible alternatives listed above, no clear alternative exists for methylcellulose at this moment that provides all functionalities needed. 

Among the plant-based meat analogues, whole-cut products (like chicken chunks) have the lowest demand for additives; the recipes are simpler and the products do not need adhesion. However, some products could benefit from the use of binders when it comes to water and fat retention, which can improve the mouthfeel and juiciness perception. Mattice and Marangoni (2020) suggested the addition of gums to improve the water holding capacity of zein fibres destined for chicken-like meat analogue formulations [163]. Possible additional ingredients for such applications are polysaccharides such as pectin, guar gum, carrageenan, cellulose, methylcellulose [177,186,187,188,189,190,191]. Polysaccharides can be also introduced during the thermomechanical processing, where they improve the rheological properties of mixtures and the water-binding capacity of meat analogues, as was observed in the case of soy/pectin based meat analogues produced in the shear cell [192]. For more natural and clean label products, however, the selection of ingredients that naturally contain polysaccharides such as protein concentrates or protein-rich flours is advised for the production of extruded or sheared products. 

### 3.3. Fat, Oil and Oil Substitutes

Fat contributes to the juiciness, tenderness and flavour release, which are important attributes for meat products [193,194]. Therefore, the addition of fat or oil in meat analogues can have similar advantages. Emulsion-type meat analogues currently available on the marked such as sausages have a fat content of up to 25%, which is similar to corresponding meat products [15]. In these products, fat is pre-emulsified and introduced together with the protein and the rest of the ingredients in the mixing stage (see Section 2.1 Emulsion-type products). This differs from burger-type analogues based on TVP, where fat is introduced through mixing the (pre-processed) ingredients, in the cold mixing step to create the final product. Muscle cut mimicking meat products could benefit from practices applied to corresponding meat products, where fats or oils are injected into the muscle to improve characteristics and consumer acceptability [195,196,197]. 

Fat should be properly balanced in the selected formulations as it influences the structure, rheological properties (fluidity, plasticity, texture) and sensory characteristics of the products [198]. Pre-emulsification of fat can be achieved by using a variety of plant-based proteins (see Section 3.1). The extraction and use of native plant oleosomes has also been suggested as an emulsifier [199]. The formulation of gels using pure oleosomes [200] or in combination with proteins [201] and carbohydrates [202] have been reported, which can be interesting for emulsion-type applications or comminuted products. For whole cut meat analogues, the use of emulsified and crosslinked fat crystal networks [30,203] and oleogels [204,205] is considered promising when aiming at a marbling structure. 

The fats and oils mostly used in meat analogue applications originate from soy, sunflower, rapeseed, canola, corn, palm, coconut and sesame oil. Interestingly, the co-products of many of them: defatted meals, flours and their derivatives (protein concentrates and isolates), are used or considered as the protein sources of meat analogues. This raises the question of whether it is necessary to fractionate these oilseeds to proteins and oils. The oilseed ingredients rich both in fat and proteins could be another way to introduce lipids into a meat analogue recipe. Ingredients such as full-fat soy flour and (naturally containing fat) mildly fractionated soy isolates have been used to produce fibrous meat analogues structures using the shear cell technology [46]. The addition of fat/oil during processing, however, can affect the structuring potential of the ingredients. When used in high quantities (>5%) during thermomechanical processing (extrusion and shearing), slip can is observed which negatively influences the shear forces and thus the fibre formation process [206]. Additionally, it was reported that extrusion of recipes containing more than 15 wt% of oil, negatively affected the alignment of the macromolecules and the fibre formation for the meat analogues, due to excessive material lubrication [207]. Thus, one can take into consideration the intended use of the material (whether this will be used in the structuring process or as a binder in the final formulation).

Abundant consumption of fat is associated with adverse health effects, which explains that part of the research on meat products, focusses on reducing the actual fat content [208,209]. Research on fat substitutes consisting of water and functional ingredients is picking up [210,211,212,213]. Fat substitutes may also be considered for the formulation of low-fat meat analogues, where juiciness, succulence and texture need improvement. Fat substitutes already used in the food industry are protein particles (from milk, eggs, or plants (i.e., soy protein isolate)), modified lipids, such as synthetic lipids (Olestra^®^ sucrose polyester); carbohydrates, including modified starches, resistant starches, dietary fibres, amorphous cellulose fibre (Z-trim^®^), etc. [214,215,216,217,218]. Among these, carbohydrates have gained a lot of attention in meat applications. Amorphous cellulose fibre has been successfully used for partial replacement of pork back fat in fermented sausages [219]. Dietary fibres such as inulin and fructooligosaccharides have been used in low quantities in low-fat Italian-type salami [220], while alpha-cyclodextrin and wheat fibre have been proposed as ingredients in chicken frankfurters [221]. Another successful example is konjac gels combined with other ingredients (starch, carrageenates, gellan gum) which have been used in the formulation of reduced/low-fat meat products such as frankfurters, bologna, fresh sausages and pork nuggets [222,223]. The positive aspects of fat substitutes do not stop on sensorial benefits. The use of rice-starch oleogels in beef burgers diminished cooking loss and fat absorption as well [205].

### 3.4. Flavour and Colouring Agents

Meat analogues acceptance is largely determined by their visual appearance and flavour. After providing the right texture and shape, the focus is on colour or colour changes during preparation that helps the product to resemble meat. Most commonly used ingredients for meat analogues, like soy protein and gluten, have a beige or yellow-brown colour. Thus, the colour of the basic meat analogue structure is far different from the well-accepted red colour of unprocessed meat or the brown colour of cooked meat. Therefore, colourants are added to the ingredient mix. The mixing can be done before any structuring treatment i.e., high-temperature shearing and extrusion (for muscle-type products), or with the rest of the ingredients in the final product formulation stage (for sausage and burger types of products). Depending on the application stage and the type of product, colouring requirements can differ. For example for sausage-type applications, heat-stable red hues are used, while for raw meat-type analogues browning or decolouring of the original red hues is desired. The difficulty in the latter case is the determination of proper red colour hues that present high storage stability at the pH value of the meat analogue but degrade or brown upon heating.

#### 3.4.1. Colouring Agents

Heat stable colouring ingredients, such as caramel colours, malt or annatto, turmin, cumin, erythrosine and carotenoids, such as carotene, canthaxanthin and lycopene [224,225,226,227,228,229], can provide the desired hues for products such as sausages or meat analogues mimicking cooked final products. These colourants are chemically synthesised or naturally derived components; the latter has gained popularity and come in the form of extracts or dry powders. Carotenoid-rich extracts like paprika oleoresins and annatto seed extracts, as well as lycopene-rich extracts from tomatoes, have been used in meat-based products, such as fermented sausages [229,230,231]. Red yeast (*Monascus purpureus*) products made by fermenting rice, are used traditionally in Chinese cuisine for the colouration of duck meat and has also been introduced in tofu and other food products [232]. Studies on natural colourants indicate that those ingredients possess antioxidant properties suggesting that they could act as nitrite replacers in meat products [229,233]. The combination of colouring agents (paprika oleoresin, lycopene and red yeast rice) has been successfully applied in “non-meat-based sausages” based on soybean isolates, gluten, soy oil, egg white, carrageenan, and modified starch [227]. Due to their stability, these colourants fit well for final product formulations. 

Meat analogues marketed as a replacer for “raw” meat products (whole-cut meat type, burgers or minced meat) require colour changes upon cooking. Therefore, heat-stable colouring agents, even of natural origin, need to be replaced by or combined with colourants that allow a colour change similar to that of meat upon cooking or frying. For meat, it is observed change from red to pink or grey-brown, due to the denaturation of myoglobin, occurs at a temperature range around 75 °C [234,235]. To mimic this, betanin and beetroot extracts are proposed as additives attributing a “raw meat” colour [225,236,237] and undergo colour changes due to thermal degradation [238]. Beetroot extract was the choice of colourant for the “raw” burger formulation by Beyond Meat™. Impossible Foods™ created a “bleeding” plant-based burger with the use of soy leghemoglobin, which is now recombinantly produced. Leghemoglobin gives their product the colour of fresh meat, while after cooking the desired browning together with meat-like aromas was observed [226,239]. Chemical changes including Maillard reactions influence the colour of the products not only during the preparation of final by the consumer but also during thermomechanical structuring (extrusion or shearing). This explains why heat-labile colourants and reducing sugars are used in various combinations depending on the meat analogue production technology and the characteristics of the final product [225]. Reducing sugars already reported in meat analogue formulation patents are dextrose, maltose, lactose, xylose, galactose, mannose and arabinose [225,236]. 

Despite the available embodiment methodologies and the variety of colourants, the final products are not always of the highest possible quality. In many applications, mismatches among the pH range of the colourant and the pH of the meat analogue are the cause of the colour problems. Therefore, acidulants, such as citric acid, acetic acid, lactic acid or their combinations are added to the formulation [240]. However, pH changes are known to alter the properties of the proteins, affecting the proteins structuring as well as the taste of the final meat analogues. Besides, proteins have a buffering capacity, thus require large quantities of additives to induced pH changes. Furthermore, along with the colouring agents colour retention aids such as maltodextrin and hydrated alginate, are used to inhibit or control the colour migration from the dyed structured meat analogue [240].

#### 3.4.2. Flavouring Agents

Like with the colouring agents, the selection of the flavour depends on the final product formulation. Starting from the selection of the plant protein ingredients, it is known that soy and legume ingredients have unpleasant flavour profiles and intrinsic off-flavours, which hinders the acceptability of the products [241]. Astringent and bitter flavours are encountered due to the presence of glycosides, like saponins, and phenols, such as isoflavones, catechins and phenolic acids [21,241]. Off-flavours such as grassy and beany flavours are formed due to lipid oxidation [242]. Practises such as defatting, removal and deactivation of lipoxygenases and even fermentation of the ingredients are suggested to remove the undesirable compounds [243].

Meat in itself has a quite neutral taste, while meat analogues mostly mimic processed meat products (see sections above). Thus, apart from correcting the off-flavours, desired aroma and taste needs to be introduced depending on whether the final product mimics marinated meat, burgers or sausages. When measuring commercial meat analogue burger products, He et al. (2021) examined the effect of cooking on aroma formation and found that the fatty acid composition and the volatile flavour substances profile are mainly determined by the flavouring ingredients used [244]. The selection of the right spices can thus increase consumer acceptance of those products. 

For flavouring of meat analogues natural savoury spices, meaty and savoury aromas are currently used [21]. Besides these, the use of precursors is explored as well [245]. Earlier research was focused on volatile components produced during the cooking of meat [246,247,248,249]. With this knowledge, meat-like flavours have been produced using precursors like reducing sugars (glucose, xylose, fructose, and ribose), amino acids (cysteine, cystine, lysine, methionine, proline, serine, threonine), vitamins (such as thiamine), nucleotides and iron complexes (e.g., ferrous chlorophyllin or heme-containing proteins) [245,250,251]. Chemical reactions, such as Maillard reactions [252,253], create new characteristic flavour substances from sugars and amino acids [254,255]. Recently, Chiang et al. (2020) explored the use of Maillard-reacted beef bone (MRP) hydrolysate to provide meat sensory aspects in extruded soy and gluten-based products [256]. Even though the added ingredient does not comply with a vegetarian and/or vegan diet, the produced flavour can increase the acceptance of meat analogues among consumers that enjoy eating conventional meat [257]. Modification of plant-based protein with the use of enzymatic hydrolysis was also found to develop desirable chicken- and beef-like aromas [258,259]. Compounds such as furans and thiophenes containing sulphur are also known to possess strong meat-like aromas with exceptionally low threshold values [260], thus cysteine/ribose reactions dominated by sulphur-containing compounds were used as contributors to an overall roasty, meat-like aroma [254,261]. Such roast aroma or flavour is the most desired, though a risk of off-flavour formation exists. Therefore, the optimization of the flavour and taste perception quality is a challenge [262,263]. Depending on the nature of these compounds, complex chemical reactions may occur, while under high temperature and pressure treatment volatile components are released from the material leading to flavour perception changes. The latter is observed during thermomechanical processing, together with moisture loss when the material leaves the extrusion die [264].

The protein ingredients used during extrusion play an important role as well. Flavour compounds, such as aldehydes, ketones, and esters, bind with proteins through hydrophobic and even through covalent interaction with cysteines [71,265,266,267]. During extrusion, changes in the volatile flavour substances are observed, associated with the microstructure of the meat analogue, the water distribution and the protein conformation [264]. Guo et al. (2020) reported that an increase in the gluten content of extruded SPI: gluten formulations lead to volatile losses and a decrease in the volatile retention rate [264]. Extrusion reduces the available binding sites for wheat gluten, among others due to disulphide cross- linking [60,264]. Thus, research on understanding how volatile flavours interact with the protein matrix can be beneficial for developing meat flavours that can be introduced during the food structuring step.

Together with aromas and precursors, salt also plays an important role in taste perception. However, the role of salt is not only as a taste enhancer, but it also contributes to the extension of the shelf life of the product. In some cases, it also improves the product’s texture. Salt addition to the protein base of the meat analogues, can lead to the solubilisation and unfolding of the protein affecting its structuring potential (see Section 3.1). It should be noted that the protein isolates contain already quite some salt due to the fractionation process [268]. The high level of sodium in the western diet and the increased health risks that it brings have raised concerns [269]. Looking for inspiration from the meat industry, sodium reduction strategies include the use of salt replacers and/or flavour enhancers. However, the sensory impact of these components is found to be matrix dependent [270].

### 3.5. Water

Water in meat analogues has multiple functions. It acts as a hydration medium for the different dried ingredients and as a plasticizer and reaction agent during processing. In extrusion processing specifically, water determines the viscosity of the melt, participates in the chemical reactions (starting with the induction of conformational changes in protein), influences the friction and acts as an energy transfer (thermal and mechanical) medium [271]. In low moisture extrusion, the moisture content together with the temperature affects the expansion and porosity of the product as it is observed for starch-containing extrudates [272]. With increasing moisture content during highly concentrated plant protein extrusion, an increase in the reaction rates of proteins is reported [273]. The disulphide bonds, hydrogen bonds and hydrophobic interactions are promoted at higher moisture levels. This can lead to a high degree of fibrous structure formation [271], while as already mentioned in the previous section interaction with the flavour components can also occur. 

In addition, many of the functional properties of the proteins as swelling, viscosity, gelation, emulsification, and foaming are affected by the availability of water in the food system and the degree of interaction with the biopolymers [274,275]. This is important for specific meat analogue application such as in sausage-type products where water is needed for the emulsification process. Moreover, the higher water content can be desirable since sensory properties such as juiciness and mouthfeel of meat analogues are retained longer. Besides, meat analogues with high water content can also be baked and cooked similarly to meat. To sustain the desired water content, a variety of binders with water-holding properties are used (see Section 3.2). Lastly, the inclusion of water in food reduces the ingredient costs. 

## 4. Steps for the Production of the Future Meat Analogues 

Consumer preference studies revealed that key motivations for people to switch towards a plant-based diet and thus include meat analogue products in their eating routine are the health benefits and the price of the products [2]. Possible constructs on the other hand are food neophobia and meat attachment [2]. This means that future research, development and innovation on meat analogue formulation and production should address all these aspects. Currently, the main focus has been given on the development of meat analogues that recreate conventional meat in all of its physical sensations (appearance, texture, taste, smell, etc.), however, there is an increasing demand by consumers for sustainable ingredient sourcing, natural, clean-label and nutritious products. 

### 4.1. Structure Formation

To achieve the desired texture and bite in current meat analogues, familiar ingredients to the consumer, such as soy, gluten and pea protein are used, however from this review we see that more and different types of plant proteins can be exploited. Mixing, heating and (low and high moisture) extrusion are still the most established texturization methods to create meat-like structures, while there is new research on novel structuring methods such as shear cell, spinning and 3D printing. For each structuring technology and product applications (emulsion-, burger- and muscle-type meat analogues), product formulations are optimised based on the protein ingredients used. Many additional ingredients (binders and texturizing agents) are added to improve the texture of the final product. 

What is known so far is that in sausage type formulation, plant proteins should answer the requirements for solubility, emulsification/fat stabilization and water binding capacities, while they should also form a firm elastic gel [15,17]. For meat analogues based on extrusion and shearing, protein-crosslinking is considered the main structuring mechanism. Properties such as water holding capacity and solubility are often regarded as good indicators of cross-link density and formation during processing, respectively [276]. However, covalent cross-links are unfavourable before or during thermomechanical processing, since they decrease chain mobility, increase viscosity, and preventing material homogenization [276], while the also affect other sensorial characteristics (Section 3.4.2 Flavouring agents). The identification of these requirements paves the way to the use of currently underutilised protein sources. 

### 4.2. Exploring Novel Proteinaceous Ingredients 

Novel protein sources are constantly explored. Proteins from crops such as rapeseed and sunflowers (leftovers from oil production) and legumes, can be considered good alternatives to soy and are expected to be implemented soon in meat analogue products [118]. The fact that they come in vast quantities and can provide high-quality proteins makes them really attractive for the food industry, while their functional properties allow their implementation in a variety of meat analogue products. Researchers aim at exploring other protein crops as well such as leaves (RubisCo) and aquatic biomasses such as duckweed, seaweed and algae, however, the technology to extract proteins from those sources is still in the initial development stage, making them less competitive towards the plant protein (examined in this review in short term [277]). There are additionally new developments on protein sources concerning recombinant or cellular agriculture, which use fungi, yeast and bacteria for the production of animal proteins or for proteins that are natural for the microorganism. However, also for this downstream processing and determination of their functional properties are still subjects to recent research [164].

In addition, there is also a trend to use classical protein sources differently. There is less focus on purity and more on functionality. This trend aligns with traditional techniques of making plant-based products such astofu, yuba and tempeh, where flours, milk or even whole beans were used as main ingredients [7]. These products show the potential of using less refined ingredients and argue even in the direction of designing or modifying protein-rich ingredients to fit specific requirements for each application. This approach can potentially reduce the product’s environmental impact [4,278,279], though the nutritional quality and the functionality of highly refined and less refined ingredients (and their by-products) should be carefully considered in such comparison [280,281]. This means that the subject of the research should be understanding when this switch can be made [18] and what the actual environmental benefit can be.

### 4.3. Functionality of Protein Sources 

The plethora of plant proteins and their properties suggests that the amino acid sequence of the protein defines the functionality [276]. The technological functionality of the proteins though can be further influenced by processing creating new possible uses for the proteins (see Section 3.1). Apart from technological functionality, plant proteins should also replace meat nutritionally, taking into consideration the amino acid profile, digestibility and bioavailability of the proteins [282,283]. An indication of composition differences between animal proteins, traditional and alternative plant-based protein sources is given in Table 2. Although some plant proteins may be considered inferior to animal proteins due to their deficiency in the essential amino acid composition and their low digestibility [284,285], a combination of ingredients to achieve a balanced amino acid profile and introduction of treatments that improve digestibility could be an option. 

However, as discussed in this review, currently available meat analogues contain apart from proteins and oils/fats (that are encountered in meat), also carbohydrates that play the role of binding agents and texturizers. This deviation in the composition can be beneficial for the target customer segment; meat-eating consumers, whose eating patterns are now characterized by protein overconsumption, due to high intake of animal-based products (meat and dairy) [286]. In this case, nutritional requirements in terms of protein intake are less strict, while consumers can benefit from a product with a diverging composition (i.e., containing a higher dietary fibre content, vitamins, minerals, antioxidants, etc.). 

## 5. Conclusions 

Creating meat analogues that mimic meat both nutritionally and in its physical sensations can increase people’s willingness to substitute (part of) their meat consumption. The latest research and development activities on meat analogues have shown that it is possible to produce meat-like texture with the use of plant-based proteins and technologies such as extrusion, shearing and mixing. However, to mimic meat in other sensorial characteristics, such as colour, aroma and mouthfeel, additional non-protein ingredients are used. The diversity in ingredient functionality requirements among the different plant-based meat analogues types (sausages, burgers and whole-cuts) does make product development complicated. 

Generally stated, the functional properties of readily available protein-rich ingredients (mostly highly purified from plant material) are not optimal for meat analogue applications. This can be seen from the necessity to use additives to improve the texture of the products. The non-optimal ingredient properties can be attributed to the fact that the ingredients used in meat analogues were designed before meat analogue applications became important. This means that there might be room for improvement of the ingredients towards meat analogue production. Novel functionality can be achieved by applying other fractionation methods, which aim for functionality rather than purity. Next to that proteins from novel sources can bring novel ingredients for meat analogue applications. 

## Figures and Tables

**Table 1:** Summary of already used protein ingredients for meat analogue applications.

Protein Ingredient	Composition (%*w*/*w*)	Functionality	Application in Meat Analogues
Soy isolate (alkaline/acid precipitation treatment)	~90 % protein	Good solubility, gelling and emulsification	Structuring process: Extrusion, shear cell, spinning, freeze structuring Role: Protein source, texture, binder, base for fat substitutes, emulsifier Products: Burger patties, minced meat, sausages
Soy isolate (additional heat treatment/ toasted isolate)	~90 % protein, denatured due to heat treatment	Decreased solubility, increased water holding capacity, good gelling	Structuring process: Extrusion, shear cell Role: Protein source, texture, binder, base for fat substitutes Products: Burger patties, minced meat, sausages
Soy concentrate	~70 % protein	Good texturization properties	Process: Extrusion, Shear cell Role: Protein source, texture, binder Products: Burger patties, minced meat, sausages, muscle-type products
Soy milk (spray-dried powder)	>45% protein, ~30 % fat	High solubility, good emulsification properties	Process: Freeze structuring Role: Emulsifier, texture Products: Tofu and yuba production
Soy flour/meal (defatted)	~43–56% protein, ~0.5-9% fat, ~3–7% crude fibre, >30% total carbohydrate	Water binding capacity and fat retention, native protein	Process: Extrusion Role: Texture, Binder Products: Burger patties, minced meat, sausages, muscle-type products
Wheat Gluten isolate	75–80% protein, 15–17% carbohydrates, 5–8% fat	Binding, Dough forming/ Cross-linking capacity via S-S bridges, low solubility	Structuring process: Extrusion, shear cell Role: Adhesion, texture Products: Burger patties, muscle-type products
Pea isolate	~85% protein	Water and fat binding, emulsification, and firm texture after thermal processing	Process: Extrusion, shear cell, spinning Role: Emulsifier, texture, Binder Products: Burger patties, minced meat, sausages, muscle-type products

**Table 2:** Composition (%) and essential amino acid contents (in g/100 g) of different plant and animal protein sources.

Composition (% )	Egg Dried	Beef **	Milk Dry Whole	Soy			Wheat Flour		Pea Seeds	Lupine Seeds	Sunflower Seed Kernels	Peanut Flour Low Fat
				Isolate	Concentrate	Flour						
Protein	48.05	21.91	26.32	83.3 ± 0.7 (N × 5.7)	63.63	37.0 ± 1.1 (N × 5.7)	9.61		23.12	36.17	20.78	33.8
Lipid	43.9	4.62	26.71	-	0.46	21.8 ± 0.4	1.95		3.89	9.74	51.46	21.9
Carbohydrates	1.13	0	38.42	13.3 ± 0.7	25.41	34.5 ± 2.2	74.48		61.63	40.37	20	31.27
Ash	4.13	1.09	6.08	3.4 +0.0	4.7	6.7 ± 0.7	1.53		2.67	3.28	3.02	5.23
Reference	[287]	[287]	[287]	[288]	[287]	[288]	[287]		[287]	[287]	[287]	[287]
**Essential amino acids**	**Recommended daily allowances (RDA in mg, for a 70-kg man)**	**Essential amino acids content in g/100 g of product**										
		**Egg**	**Beef**	**Milk**	**Soy isolate**	**Soy concentrate**	**Defatted soy flour**	**Wheat flour**	**Pea seeds**	**Lupine seeds**	**Sunflower Seed kernels**	**Peanut flour low fat**
Histidine	700	1.202	0.699	0.714	2.303	1.578	1.268	1.4	0.586	1.03	0.632	0.854
Isoleucine	1400	2.434	0.997	1.592	4.253	2.942	2.281	2.0	0.983	1.615	1.139	1.188
Leucine	2730	4.15	1.743	2.578	6.783	4.917	3.828	5.0	1.68	2.743	1.659	2.191
Lysine	2.100	3.339	1.852	2.087	5.327	3.929	3.129	1.1	1.771	1.933	0.937	1.213
Methionine	1050 *	1.495	0.571	0.66	1.13	0.814	0.634	0.7	0.195	0.255	0.494	0.415
Phenylalanine	1750 *	2.53	0.865	1.271	4.593	3.278	2.453	3.7	1.151	1.435	1.169	1.752
Tryptophan	280	0.775	0.144	0.371	1.116	0.835	0.683	Not measured	0.159	0.289	0.348	0.328
Threonine	1050	2.129	0.875	1.188	3.137	2.474	2.042	1.8	0.813	1.331	0.928	1.158
Valine	1820	2.991	1.087	1.762	4.098	3.064	2.346	2.3	1.035	1.51	1.315	1.418
Reference	[289]	[287]	[287]	[287]	[287]	[287]	[287]	[290]	[287]	[287]	[287]	[287]

* These values are reported as the sum of cysteine and methionine, and phenylalanine and tyrosine ** Beef, top sirloin, steak, separable lean only, trimmed to 1/8” fat, choice, raw.
